# Oxidative phosphorylation-dependent regulation of cancer cell apoptosis in response to anticancer agents

**DOI:** 10.1038/cddis.2015.305

**Published:** 2015-11-05

**Authors:** N Yadav, S Kumar, T Marlowe, A K Chaudhary, R Kumar, J Wang, J O'Malley, P M Boland, S Jayanthi, T K S Kumar, N Yadava, D Chandra

**Affiliations:** 1 Department of Pharmacology and Therapeutics, Roswell Park Cancer Institute, Buffalo, NY 14263, USA; 2 Department of Biostatistics and Bioinformatics, Roswell Park Cancer Institute, Buffalo, NY 14263, USA; 3 Department of Medicine, Roswell Park Cancer Institute, Buffalo, NY 14263, USA; 4 Department of Chemistry and Biochemistry, University of Arkansas, Fayetteville, AR 72701, USA; 5 Pioneer Valley Life Sciences Institute, Springfield, MA 01107, USA

## Abstract

Cancer cells tend to develop resistance to various types of anticancer agents, whether they adopt similar or distinct mechanisms to evade cell death in response to a broad spectrum of cancer therapeutics is not fully defined. Current study concludes that DNA-damaging agents (etoposide and doxorubicin), ER stressor (thapsigargin), and histone deacetylase inhibitor (apicidin) target oxidative phosphorylation (OXPHOS) for apoptosis induction, whereas other anticancer agents including staurosporine, taxol, and sorafenib induce apoptosis in an OXPHOS-independent manner. DNA-damaging agents promoted mitochondrial biogenesis accompanied by increased accumulation of cellular and mitochondrial ROS, mitochondrial protein-folding machinery, and mitochondrial unfolded protein response. Induction of mitochondrial biogenesis occurred in a caspase activation-independent mechanism but was reduced by autophagy inhibition and p53-deficiency. Abrogation of complex-I blocked DNA-damage-induced caspase activation and apoptosis, whereas inhibition of complex-II or a combined deficiency of OXPHOS complexes I, III, IV, and V due to impaired mitochondrial protein synthesis did not modulate caspase activity. Mechanistic analysis revealed that inhibition of caspase activation in response to anticancer agents associates with decreased release of mitochondrial cytochrome *c* in complex-I-deficient cells compared with wild type (WT) cells. Gross OXPHOS deficiencies promoted increased release of apoptosis-inducing factor from mitochondria compared with WT or complex-I-deficient cells, suggesting that cells harboring defective OXPHOS trigger caspase-dependent as well as caspase-independent apoptosis in response to anticancer agents. Interestingly, DNA-damaging agent doxorubicin showed strong binding to mitochondria, which was disrupted by complex-I-deficiency but not by complex-II-deficiency. Thapsigargin-induced caspase activation was reduced upon abrogation of complex-I or gross OXPHOS deficiency whereas a reverse trend was observed with apicidin. Together, these finding provide a new strategy for differential mitochondrial targeting in cancer therapy.

Cancer cells favor glycolysis over oxidative phosphorylation (OXPHOS) to meet their energy demand,^1^ suggesting that they have adapted to survive and proliferate in the absence of fully functional mitochondria. Research in the last two decades demonstrates that, in addition to generation of energy, mitochondria including cancer cell mitochondria regulate multiple cellular signaling pathways encompassing cell death, proliferation, cellular redox balance, and metabolism.^2, 3^ As cancer cells possess defects in these pathways that provide an opportunity to target this organelle for therapeutic purposes. Subsequently, several agents have been developed that target cancer cell mitochondria to induce apoptosis, a cell death pathway, and eradicate cancer cells.^4, 5^ Cancer cell mitochondria harbor several proapoptotic proteins including cytochrome *c*, which is released from mitochondria in response to anticancer agents and activates caspases to execute apoptosis.^5, 6^ Thus, anticancer agents that induce cytochrome *c* release from mitochondria will be beneficial for induction of apoptosis in cancer cells. Indeed, several such agents have been developed, which include inhibitors targeting prosurvival Bcl-2 family members including Bcl-2, Bcl-xL, and Mcl-1.^7, 8, 9^ Unfortunately, cancer cells have developed multiple mechanisms to resist or overcome cytochrome *c* release and evade apoptosis.

Although underlying mechanisms of cancer cell resistance to apoptosis are still undefined, the OXPHOS defect is known to be one of the key reasons for the attenuation of apoptosis in cancer cells.^10, 11^ Multiple lines of evidence support the notion that cancer cell survival and proliferation commonly associate with an OXPHOS defect in cancer.^1, 12^ Active OXPHOS is an efficient form of respiration but also regulates apoptosis through the OXPHOS complexes. The OXPHOS system consists of five multimeric protein complexes (I, II, III, IV, and V). The components of these complexes (except complex-II) are encoded by both mitochondrial DNA (mtDNA) and nuclear DNA (nDNA).^12, 13^ Thus mutations, deletions, and translocations in either mtDNA or nDNA can potentially result in OXPHOS deficiency. MtDNA mutations associate with inhibition of apoptosis, induction of angiogenesis, invasion and metastasis of various types of cancer.^3, 12, 14^ Thus, mtDNA could potentially be an important target to restore cell death in cancer and attenuate cancer growth. Therefore, there is an urgent need to investigate the role of OXPHOS in the molecular mechanisms underlying cancer cell death.

We investigated the effects of several anticancer agents of different classes including DNA-damaging agents (etoposide and doxorubicin), protein kinase inhibitors (staurosporine and sorafenib), mitotic inhibitor (taxol), ER stressor/inhibitor of Ca^2+^-ATPases (thapsigargin), and histone deacetylase (HDAC) inhibitor (apicidin) on mtDNA. We also determined the impact of OXPHOS defects on apoptosis induction by these agents. Although most anticancer agents induced caspase activation and apoptosis, the mtDNA level was elevated maximally by etoposide and it was not modulated by a caspase inhibitor but reduced by an autophagy inhibitor. Induction of mtDNA is associated with increased reactive oxygen species (ROS) production and elevated mitochondrial mass. Pharmacologic inhibition of OXPHOS complexes reduced the etoposide-induced elevation in mtDNA, suggesting the involvement of these complexes in etoposide-induced apoptosis. Together, we define the impact of mtDNA and OXPHOS function on mitochondrial apoptosis, which has significance in restoring cancer cell apoptosis for therapeutic purposes.

## Results

### Diverse anticancer agents induce caspase-dependent apoptosis in cancer cells

**Figure 1:**
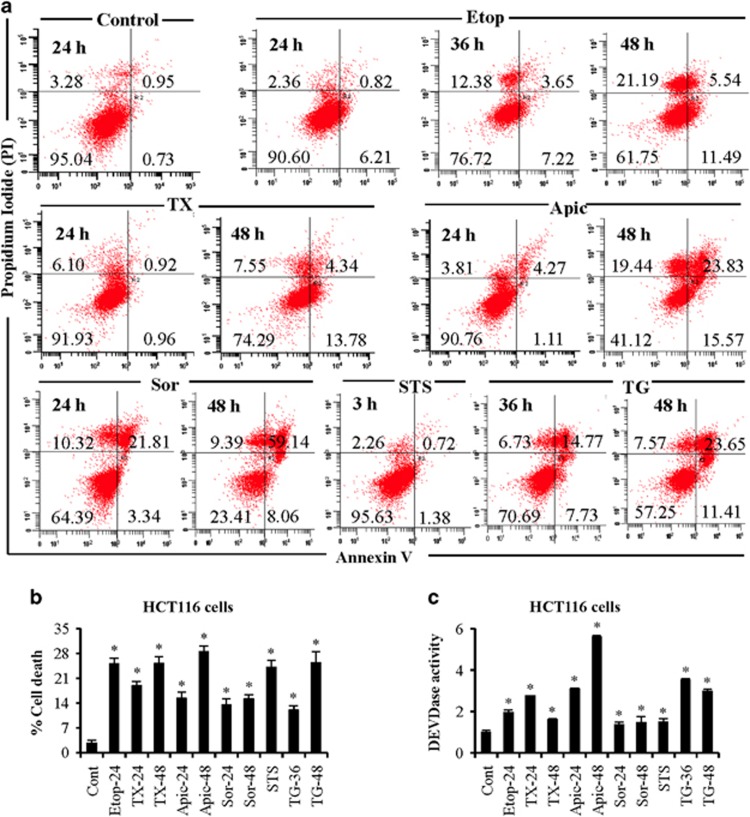
Differential sensitivity of prostate and colon cancer cells in response to multiple anticancer agents. (**a**) LNCaP prostate cancer cells were treated with etoposide (Etop; 10 *μ*M), taxol (TX; 30 nM), apicidin (Apic; 1 *μ*M), sorafenib (Sor; 20 *μ*M), staurosporine (STS; 500 nM), and thapsigargin (TG; 5 *μ*M) for various time intervals. Cells were labeled with annexin V/PI as per manufacturer's instructions and early and late apoptosis were quantified by flow cytometry analysis. A minimum of 10 000 events was collected for each sample. (**b**) HCT116 WT colon cancer cells were treated with etoposide (Etop), taxol (TX), apicidin (Apic), sorafenib (Sor), staurosporine (STS), and thapsigargin (TG) similar to LNCaP cells for various times. Total cell death was quantified using Trypan blue staining. (**c**) Caspase-3 activity was measured at excitation 400/430 nm and emission 508/520 nm using DEVD-AFC as a substrate and data are presented as fold change compared with control. Data are mean±S.D., *n*=3. *P⩽0.05 compared with control

To understand the underlying mechanism of cell death in response to multiple anticancer agents, we quantified cell death in response to etoposide, staurosporine, taxol, thapsigargin, apicidin, and sorafenib in colon (HCT116) and prostate (LNCaP) cancer cells. Etoposide, taxol, apicidin, sorafenib, staurosporine, and thapsigargin-induced apoptotic cell death in LNCaP cells (Figure 1a and Supplementary Figure S1). These anticancer agents also induced nonapoptotic cell death such as necrosis. The percentage cell death in HCT116 cells at 24 h were 22, 19, 15, 13, 24, and 12% in response to etoposide, taxol, apicidin, sorafenib, staurosporine, and thapsigargin, respectively (Figure 1b). These findings suggest that anticancer agents exert differential sensitivities and show cell type-dependent effects. Increased DEVDase activity, representing caspase-3/7 activities,^15, 16^ on treatment with anticancer agents suggests caspase-dependent apoptosis (Figure 1c). Similarly, we observed increased caspase-3 activity in PC3 prostate cancer cells and MIA PaCa-2 pancreatic cancer cells in response to doxorubicin and etoposide (Supplementary Figure S2). Together, DNA-damaging agent, protein kinase inhibitors, mitotic inhibitor, ER stressor, and HDAC inhibitor induce caspase-dependent apoptosis in cancer cells.

### Etoposide, apicidin, and sorafenib enhance mtDNA level in cancer cells

**Figure 2:**
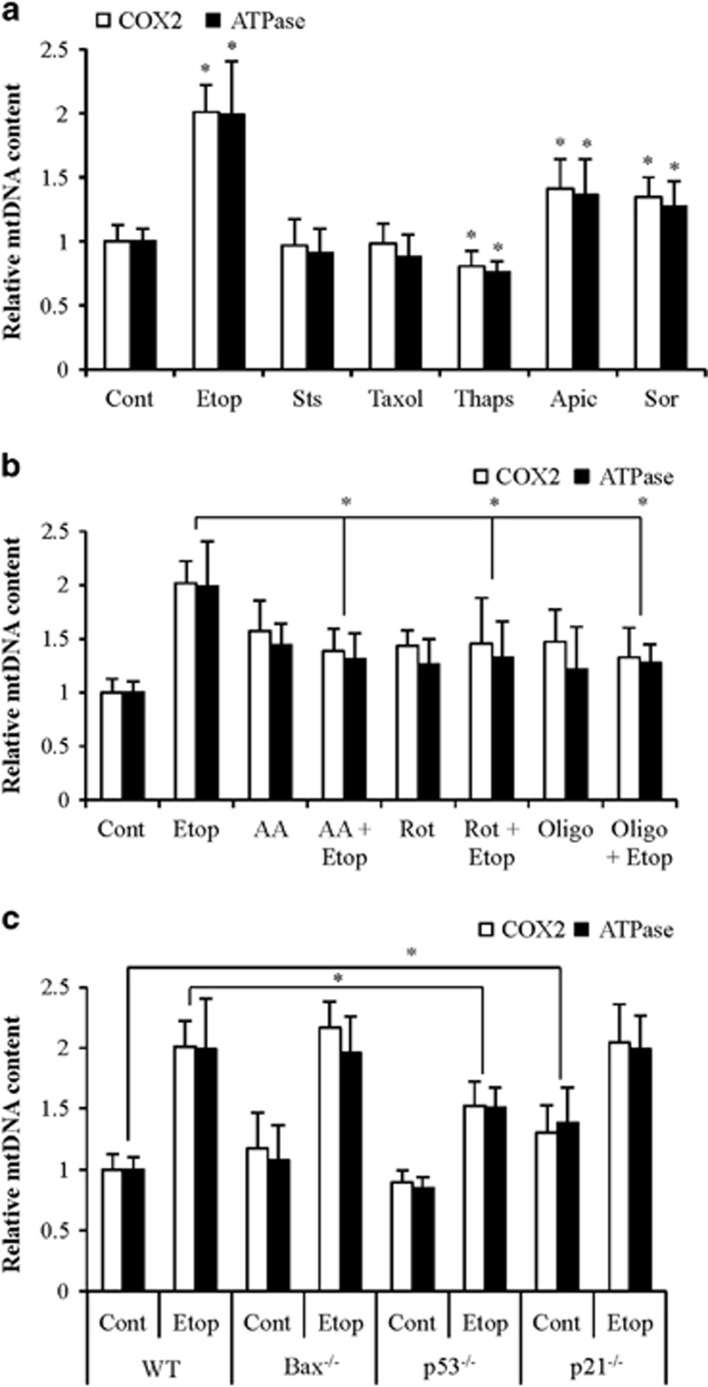
Effect of anticancer agents on mtDNA copy number. (**a**) HCT116 cells were treated with etoposide (Etop; 10 *μ*M for 24 h), taxol (TX; 30 nM for 24 h), apicidin (Apic; 1 *μ*M for 24 h), sorafenib (Sor; 20 *μ*M for 24 h), staurosporine (STS; 500 nM for 3 h), and thapsigargin (Thaps; 5 *μ*M for 24). (**b**) HCT116 cells were pretreated with OXPHOS complex inhibitors for 2 h followed by addition of etoposide (Etop; 10 *μ*M for 24 h). Etoposide-induced mtDNA levels were evaluated on inhibition of complex-I by rotenone (Rot; 10 *μ*M), complex-III by antimycin A (AA; 10 *μ*M), and complex V by oligomycin (Oligo; 10 *μ*M). (**c**) The level of mtDNA in the HCT116 Bax^−/−^, HCT116 p53^−/−^, and HCT116 p21^−/−^ cells treated with etoposide (Etop; 10 *μ*M for 24 h). Relative mtDNA level following exposure to various agents was measured by estimation of COX-2 and ATPase genes normalized to nDNA. Data are mean±S.D., *n*=4. *P≤0.05

Mitochondrial function is important for the induction of apoptosis and mtDNA is also critical for mitochondrial biogenesis.^5, 7, 17^ Evaluation of changes in mtDNA level by measuring mtDNA-encoded cytochrome *c* oxidase II and ATPase 8 gene levels normalized with nDNA encoded actin demonstrated an increased mtDNA content in response to etoposide, apicidin and sorafenib treatments; but not with staurosporine, taxol, and thapsigargin treatments (Figure 2a).

Increased mtDNA on treatment with anticancer agents may be a response to impaired OXPHOS. We observed increased mtDNA content on inhibition of complex-I, complex-III, and complex-V by rotenone, antimycin A, and oligomycin, respectively (Figure 2b). The etoposide-induced mtDNA levels were significantly attenuated in the presence of rotenone, antimycin A, and oligomycin (Figure 2b). Thus etoposide, apicidin, and sorafenib increase mtDNA content during apoptosis, whereas staurosporine, taxol, thapsigargin did not show upregulation of mtDNA.

### Bax deficiency has no effect but p53-deficiency attenuated etoposide-induced mtDNA increase

TP53 and Bax proteins regulate mitochondrial respiration, and reduction of mtDNA associates with reduced level of p53 protein.^18, 19, 20^ Indeed, we observed that p53-deficiency but not Bax-deficiency inhibited etoposide-induced mtDNA increase. The p53 target protein p21 did not modulate etoposide-induced mtDNA copy number (Figure 2c). Increased expression of antioxidant genes via the p21-signaling pathway suppresses oxidative stress.^21^ Therefore, p21-deficiency leads to modest increase in endogenous ROS causing elevated mtDNA synthesis.^22^ These findings suggest that p53 modulates mtDNA copy number and thus OXPHOS functions in response to anticancer agents.

### Etoposide induces ROS production and increases mitochondrial mass

**Figure 3:**
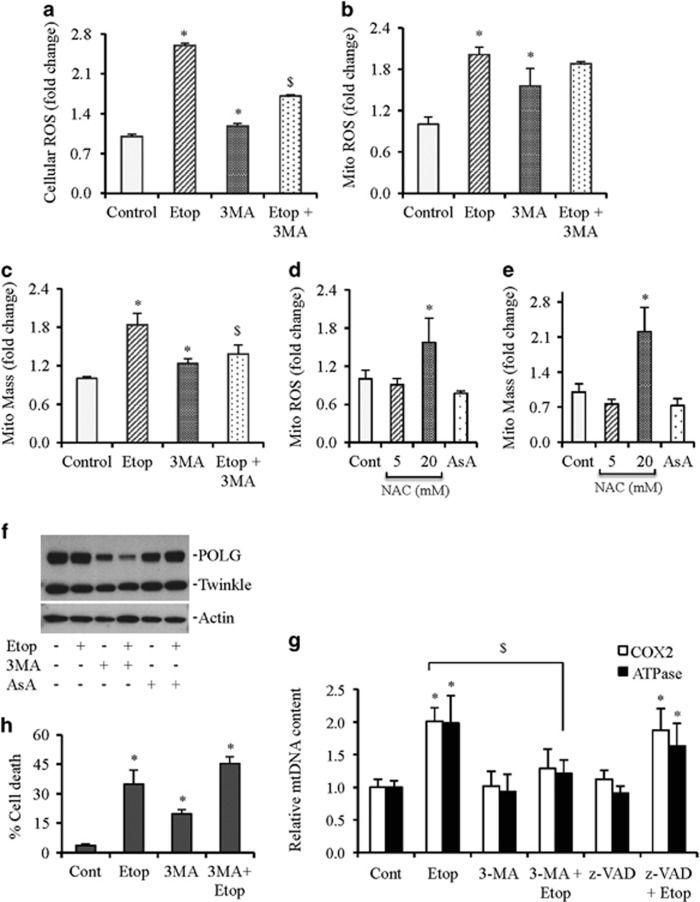
Increased ROS production and mitochondrial mass (mito mass) in response to etoposide treatment. (**a–c**) HCT116 cells were treated with etoposide (Etop; 10 *μ*M) alone or pretreated with 3MA (5 mM) followed by etoposide treatment for 24 h. Cellular ROS, mitochondrial ROS, and mitochondrial mass were estimated by flow cytometry analysis of dihydrorhodamine 123 (DHR123), MitoSox Red, and MitoTracker green, respectively. (**d** and **e**) HCT116 cells were treated with NAC (5 and 20 mM for 24 h) or ascorbic acid (AsA; 500 nM for 24 h) and mitochondrial ROS (Mito ROS) and mitochondrial mass (Mito Mass) were estimated by flow cytometry. (**f**) HCT116 cells were treated with etoposide (Etop; 10 *μ*M) alone or pretreated with 3MA (5 mM) or ascorbic acid (AsA; 500 nM) followed by etoposide treatment for 24 h. Whole cell lysates were subjected to western blot analysis. Actin serves as a loading control. (**g**) Relative mtDNA contents in the presence and absence of autophagy inhibitor 3 MA (5 mM) and pan caspase inhibitor z-VAD (50 *μ*M) followed by etoposide (Etop; 10 *μ*M for 24 h) exposure. (**h**) HCT116 cells were treated with etoposide (Etop; 10 *μ*M) alone or pretreated with 3MA (5 mM) followed by etoposide treatment for 24 h. Total cell death was quantified using Trypan blue method. Data are mean±S.D., *n*=3. *P⩽0.05 compared with control. ^$^*P*⩽0.05 compared with etoposide (Etop) treatment

ROS play an important role in mitochondrial biogenesis.^22, 23^ We observed that both cellular and mitochondrial ROS were elevated on etoposide treatment (Figures 3a and b). To evaluate whether increased ROS production leads to increased mitochondrial biogenesis, we measured mitochondrial mass on etoposide treatment. Indeed, increased mitochondrial ROS production was associated with elevated mtDNA and mitochondrial mass (Figures 3a–c).

To validate that increased mitochondrial ROS causes mitochondrial biogenesis, cells were treated with ROS scavengers *N*-acetyl-L-cysteine (NAC) and ascorbic acid to measure levels of mitochondrial ROS and mass. Ascorbic acid and lower concentration of NAC (5 mM) reduced the endogenous levels of mitochondrial ROS that was also associated with decreased mitochondrial mass. Higher concentration of NAC (20 mM) enhanced mitochondrial ROS and mitochondrial mass. Polymerase-*γ* (POLG), the only mitochondria polymerase, and mitochondrial helicase twinkle have important roles in mtDNA replication.^24, 25^ A modest decrease in the expression of POLG in response to ascorbic acid treatment suggests that ROS regulate mitochondrial biogenesis (Figures 3d–f).

### Cell death inhibitors differentially affect etoposide-induced ROS and mitochondrial mass

Increased ROS production associates with increased autophagy and apoptosis.^26, 27^ We observed that prior inhibition of apoptosis by the pan caspase inhibitor z-VAD-FMK did not prevent an increase in mtDNA level induced by etoposide (Figure 3g). In contrast, autophagy inhibitor 3-methyladenine (3MA) reduced the etoposide-induced cellular ROS. Although etoposide-induced mitochondrial ROS was reduced in response to pretreatment with 3MA but was not significant (Figure 3b), the etoposide-induced mitochondrial mass and mtDNA levels were significantly reduced in the presence of 3MA (Figures 3c and g).

Autophagy removes defective mitochondria within cells to maintain cellular homeostasis.^28^ Therefore, removal of defective mitochondria via autophagy may lead to increased mitochondrial biogenesis to cope with the stress. Hence, inhibition of autophagy by 3MA will result in accumulation of defective mitochondria, which may inhibit mitochondrial biogenesis, and activate cell death pathways.^29, 30^ Indeed, the combined treatment of 3MA and etoposide induced an increase in cell death that might have contributed to the reduction in mtDNA content (Figures 3g and h).

To provide evidence that inhibition of autophagy leads to decreased mitochondrial biogenesis or replication of mtDNA, we measured POLG and twinkle protein expression. We observed that inhibition of autophagy reduced the expression of POLG. This decrease was even more drastic when autophagy was inhibited in combination with etoposide treatment (Figure 3f). Since the majority of ROS are generated in the mitochondrial compartment, it is likely that an acute accumulation of ROS in the mitochondrial compartment in response to etoposide may lead to imbalanced redox with subsequent activation of unfolded protein response (UPR).^31^

### Upregulation of UPR during DNA-damage-induced apoptosis

**Figure 4:**
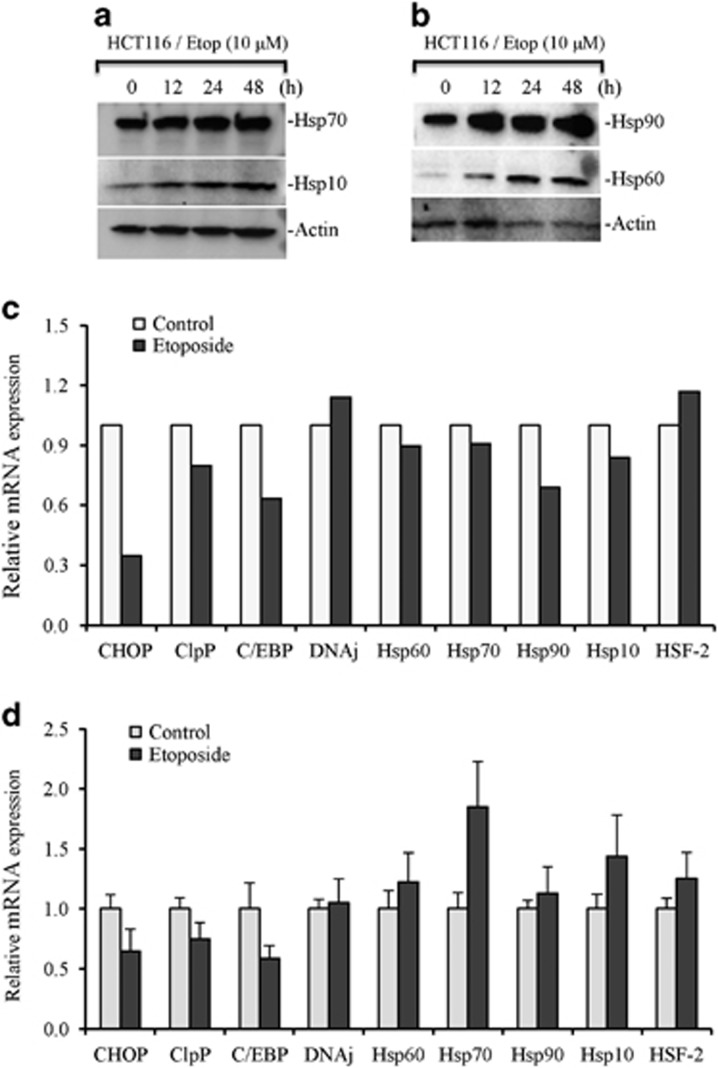
Etoposide induces mitochondrial unfolded protein response (UPR^mt^). HCT116 cells were treated with etoposide (Etop; 10 *μ*M) for various time periods. (**a** and **b**) Whole cell lysates were prepared and subjected to western blotting for mentioned mitochondrial UPR proteins. Actin serves as loading control. (**c**) HCT116 cells treated with etoposide (Etop; 10 *μ*M) for 24 h. Equal amounts of total RNA were subjected to RNA-Seq library preparation followed by sequence reads (80 millions). Expression levels of CHOP, CEBP, ClpP, DnaJ, Hsp10, Hsp60, Hsp70, Hsp90, and HSF-2 are presented. (**d**) HCT116 cells treated with etoposide (Etop; 10 *μ*M) for 24 h. Total RNAs were isolated from treated and untreated cells and equal amounts of RNA was used for real-time PCR analysis for mentioned UPR genes including the expression levels of CHOP, CEBP, ClpP, DnaJ, Hsp10, Hsp60, Hsp70, Hsp90, and HSF-2. Data are mean±S.D., *n*=3

To determine if etoposide treatment leads to defective mitochondrial protein-folding machinery, we measured the expression of multiple proteins required during the UPR including Hsp60, Hsp10, and Hsp70. We observed increased upregulation of proteins involved in mitochondrial protein-folding machinery including Hsp60 chaperonin. The expression of Hsp90, Hsp70, Hsp60, Hsp10, HSF-2, CHOP, C/EBP, ClpP, DnaJ was analyzed by RNA-Seq. Levels of CHOP, C/EBP, and ClpP were reduced upon etoposide treatment. Real-time quantitative-PCR analysis further validated decreased expression of CHOP, C/EBP, and ClpP. However, we observed an increase in the levels of Hsp70 mRNA (Figures 4a–d). Proteins destined to mitochondria have shorter untranslated regions, which give them selective advantage during translation.^32^ Therefore, increased translation efficiency of mitochondrial UPR (UPR^mt^)-regulated proteins could be a compensatory mechanism under stress causing decrease in their transcripts.

### Heat-shock response and OXPHOS/metabolism in response to DNA-damaging agents

**Figure 5:**
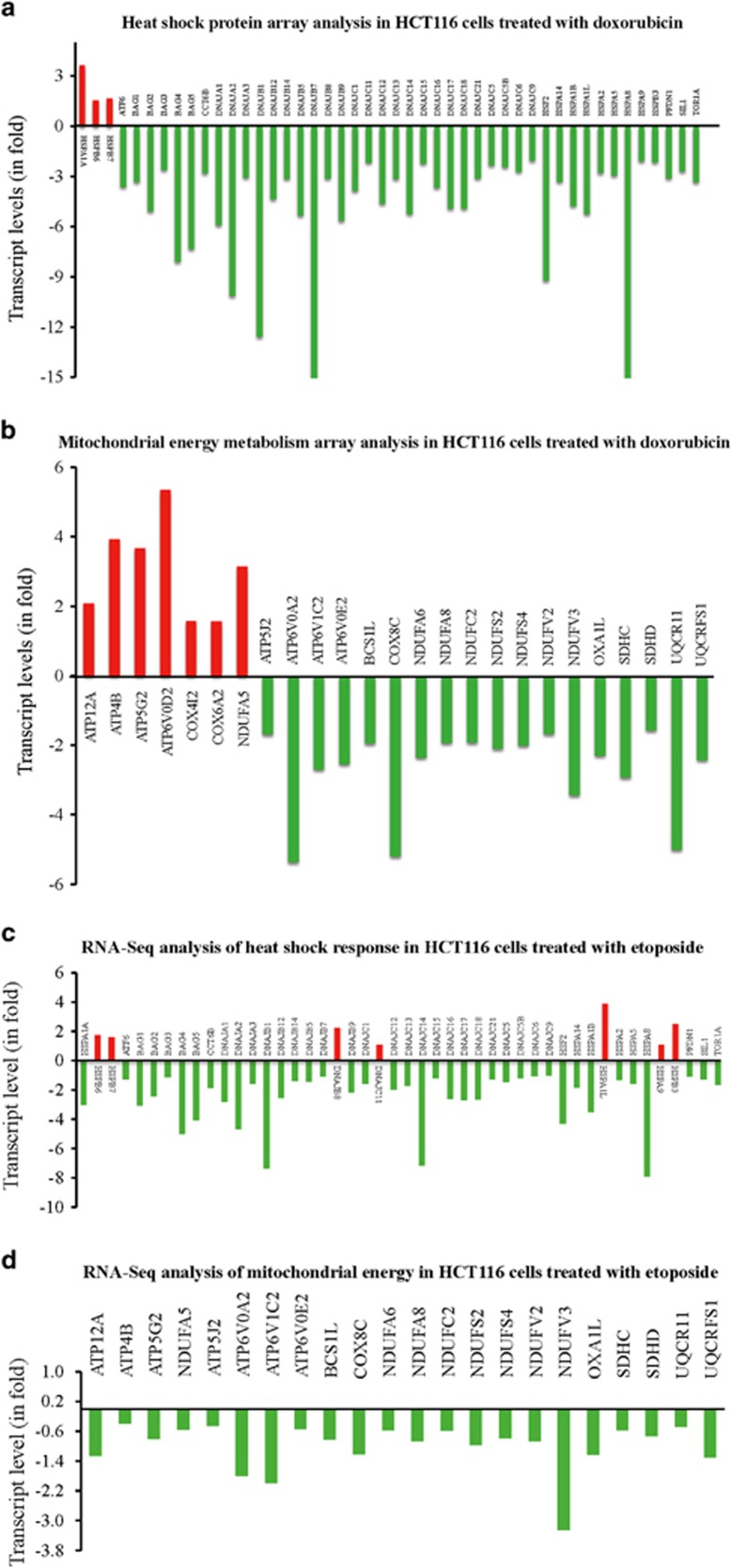
DNA-damaging agents modulate the expression of heat-shock proteins (Hsps) and mitochondrial energy proteins. HCT116 WT cells treated with doxorubicin (Dox; 10 *μ*M) for 12 h were harvested in TriZol and total RNA was isolated. 2 *μ*g of total RNA was used to convert to cDNA. Control and treated cDNAs were applied to heat-shock protein array (**a**) and mitochondrial energy metabolism array (**b**) and run on Applied Biosystems 7300 platform. Fold change relative to untreated control is presented. An average of five housekeeping genes were used to normalize the expression. (**c** and **d**) Equal amounts of total RNA were subjected to RNA-Seq library preparation followed by sequence reads (80 million). Data are presented as total transcript levels for heat-shock response (**c**) or OXPHOS (**d**) genes compared with control housekeeping genes

DNA-damaging agent etoposide affects mtDNA content and protein-folding machinery. In response to doxorubicin, we observed that of the 84 transcripts tested, 43 were downregulated at least two-fold on the heat-shock protein array, and only 3 transcripts were upregulated at least two-fold compared with vehicle treated control. Analysis of genes involved in mitochondrial energy generation showed that out of the 84 genes tested, 7 were upregulated by at least 50% as compared with vehicle treated control and 18 genes were downregulated by at least 50% in response to doxorubicin as compared with the vehicle treated control (Figures 5a and b). Thus in response to doxorubicin, UPR^mt^ was modulated in cancer cells.

Similar to doxorubicin, etoposide also reduced the expression of a majority of heat-shock proteins important for UPR^mt^. Although some differences were observed between etoposide and doxorubicin treated cells, the overall trend was similar (Figures 5a and c). Interestingly, OXPHOS transcripts were downregulated in response to etoposide, which was different from doxorubicin treatment where seven of the 84 transcripts were upregulated (Figure 5d).

### Complex-I-deficiency inhibits DNA-damage-induced caspase activation

**Figure 6:**
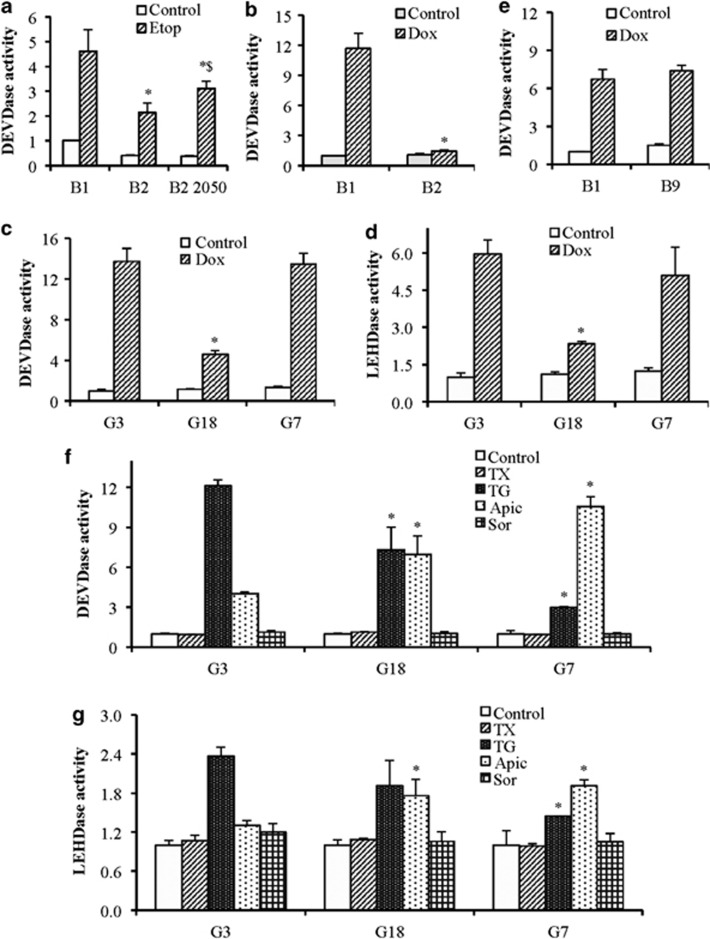
Abrogation of OXPHOS complexes modulates caspase activities. (**a** and **b**) WT (B1) and Complex 1 deficient (B2) cells or complex-I reconstituted to 50% (B2-2050) cells were treated with etoposide (Etop; 10 *μ*M for 24 h) or doxorubicin (Dox; 10 *μ*M for 24 h). (**c** and **d**) WT (G3), complex-I-deficient (G18), and gross OXPHOS deficiency (G7) cells were treated doxorubicin (Dox; 10 *μ*M for 24 h). (**e**) WT (B1) and complex-II-deficient (B9) cells were treated with doxorubicin (Dox; 10 *μ*M for 24 h). (**f** and **g**) WT (G3), gross OXPHOS deficiency (G7), and complex-I-deficient (G18) cells were treated with taxol (TX; 30 nM), thapsigargin (TG; 5 *μ*M), apicidin (Apic; 1 *μ*M), and sorafenib (Sor; 20 *μ*M). Caspase-3 (DEVDase) and caspase-9 (LEHDase) activities were measured by excitation 400/430 nm and emission 508/520 nm using DEVD-AFC and LEHD-AFC substrate for caspase-3 and caspase-9, respectively. Results are presented as fold change compared with control. Data are mean±S.D., *n*=3. *P⩽0.05 compared with respective drug-treated control B1 or G3 cells

To understand the role of OXPHOS in determining sensitivity to etoposide and other anticancer agents, we used cells lacking complex-I, complex-II, and mitochondrial protein synthesis (gross OXPHOS deficiency due to lack of mtDNA-encoded subunits of complexes-I, -III, -IV, and -V) and measured caspase activation after treatment with anticancer agents. Etoposide-induced robust caspase activation in wild type B1 cells, whereas it was reduced significantly in complex-I-deficient B2 cells. To further validate the importance of functional complex-I in etoposide-induced apoptosis, we treated *Ndufa1* reconstituted (~50% B2-2050) cells with etoposide and observed significant restoration of caspase activation (Figure 6a), suggesting that respiratory complex-I has an important role in etoposide-induced cell death.

Doxorubicin-induced caspase activation was also drastically reduced in complex-I-deficient B2 cells (Figure 6b). Similarly, wild type G3 cells also showed robust caspase-9 and caspase-3 activities in response to doxorubicin, whereas complex-I-deficient G18 cells showed drastically reduced caspase activities. Surprisingly, the caspase activation in response to doxorubicin was not impaired in G7 cells with gross OXPHOS deficiency or in B9 cells with complex-II-deficiency (Figures 6c–e).

Similar to etoposide and doxorubicin, thapsigargin and apicidin also induced caspase-3 activity in wild type G3 cells. The thapsigargin-induced caspase activation was higher compared with apicidin. However, the effect of complex-I-deficiency was opposite on thapsigargin- *versus* apicidin-induced caspase activation. Gross OXPHOS deficiency (G7 cells), as well as complex-I-deficiency (G18 cells) significantly reduced the levels of caspase activation in response to thapsigargin, whereas apicidin further enhanced the caspase activation in complex-I and gross OXPHOS-deficient cells compared with WT cells. In contrast to etoposide, apicidin, and thapsigargin; taxol and sorafenib failed to induce caspase activation even in WT cells. Similar trends were observed with caspase-9 activation (Figures 6f and g).

### Complex-I-deficiency inhibits the release of cytochrome *c* and apoptosis-inducing factor (AIF) causing inhibition of apoptotic cell death

**Figure 7:**
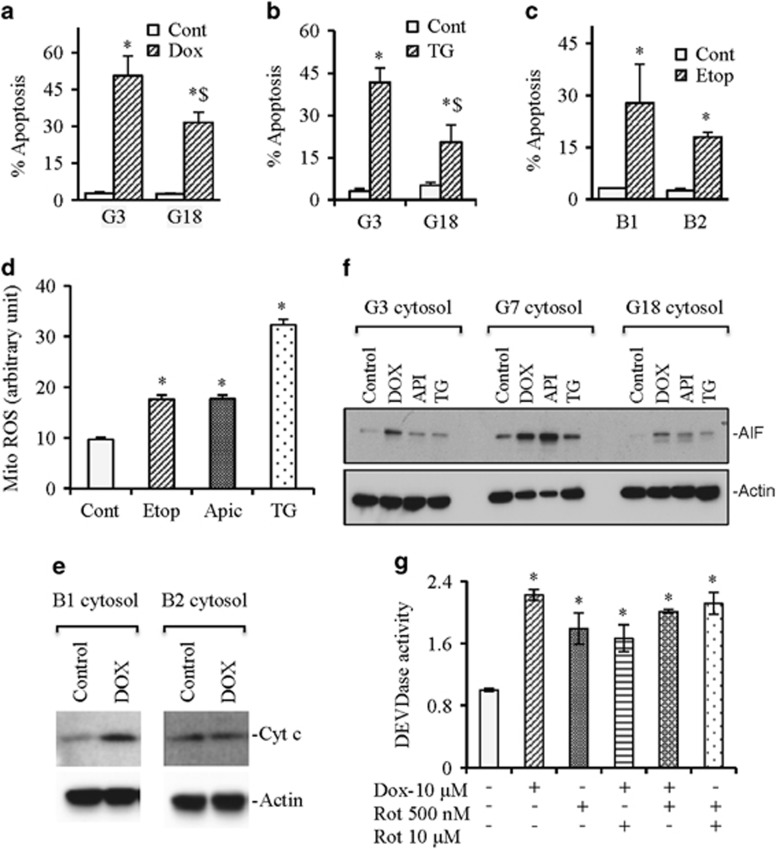
Abrogation of OXPHOS complexes modulates apoptotic cell death. (**a** and **b**) WT (G3) and complex 1 deficient (G18) cells were treated with doxorubicin (Dox; 10 *μ*M for 24 h) or thapsigargin (TG). (**c**) WT (B1) and complex 1 deficient (B2) cells were treated with etoposide (Etop; 10 *μ*M for 24 h). At the end of treatment (**a–c**), apoptosis were quantified by flow cytometry analysis. A minimum of 10 000 events was collected for each sample. (**d**) WT (G3) cells were treated with etoposide (Etop; 10 *μ*M for 24 h), apicidin (Apic; 1 *μ*M), and thapsigargin (TG; 5 *μ*M) followed by mitochondrial ROS determination using flow cytometer. (**e**) WT (B1) and complex 1 deficient (B2) cells were treated with doxorubicin (Dox; 10 *μ*M for 24 h). Cytosols were purified and used for western blotting for cytochrome *c* (Cyt c) and actin to determine the level of release Cyt c in the cytosol. (**f**) WT (G3), complex-I-deficient (G18), and gross OXPHOS deficiency (G7) cells were treated doxorubicin (Dox; 10 *μ*M for 24 h), apicidin (Apic; 1 *μ*M), and thapsigargin (TG; 5 *μ*M). Cytosols were purified and were used for western blotting for AIF and actin. (**g**) HCT116 cells were treated with doxorubicin (Dox; 10 *μ*M for 24 h) alone or pretreated with rotenone (Rot; 500 nM or 10 *μ*M) followed by doxorubicin treatment. Whole cell lysates were used for DEVDase activity measurements. Data are mean±S.D., *n*=3. *P⩽0.05 compared with respective drug-treated control B1 or G3 or HCT116 cells. ^$^*P*⩽0.05 compared with respective drug treatments

To investigate whether inhibition of caspase activation in response to anticancer agents also translates into reduction of apoptosis on complex-I-deficiency, we treated WT G3 and complex-I-deficient G18 cells with doxorubicin and thapsigargin followed by quantification of apoptosis. We observed significant reduction of apoptosis in complex-I-deficient cells compared with WT cells. Similarly, decreased apoptosis was observed in complex-I-deficient B2 cells compared with WT B1 cells in response to etoposide (Figures 7a–c).

To understand the molecular mechanism of apoptosis induction, we measured mitochondrial ROS levels in response to anticancer agents. We observed that etoposide, apicidin, and thapsigargin significantly increased mitochondrial ROS production (Figure 7d). The increased mitochondrial ROS may ultimately destabilize mitochondrial membrane leading to cytochrome *c* release causing caspase activation and apoptosis. Indeed, we observed accumulation of cytochrome *c* in the cytosol on doxorubicin treatment in WT but not in complex-I-deficient cells (Figure 7e). To understand whether caspase-independent apoptosis also has a role in response to these anticancer agents, we isolated cytosol and mitochondria to measure the release of AIF. We observed relatively increased AIF release in gross OXPHOS-deficient G7 cells compared with WT G3 cells and complex-I-deficient G18 cells in response to anticancer agents (Figure 7f).

We next determined whether inhibition of electron transport function of complex-I by its known inhibitor rotenone modulates caspase activation. Rotenone itself enhanced caspase activation, which did not show significant additive effect with doxorubicin treatment (Figure 7g). Our findings suggest that lack of subunit (for example, *Ndufa1 or Ndufb11)* causes defective complex-I assembly that leads to inhibition of apoptosis.

### Doxorubicin binds to mitochondria via complex-I

**Figure 8:**
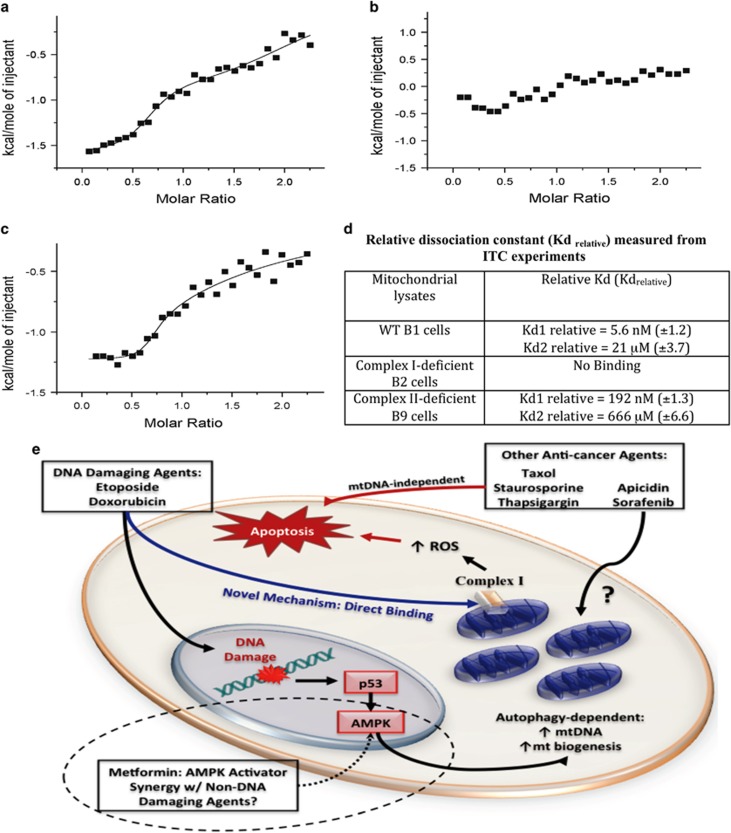
Doxorubicin binds with mitochondria in complex-I-dependent manner. Purified mitochondria from WT (B1), complex-I-deficient (B2), and complex-II-deficient (B9) cells were used for determining binding affinity with doxorubicin. Isothermogram representing the binding of doxorubicin to mitochondrial lysate suspended in 10 mM sodium phosphate buffer containing 100 mM NaCl, pH 7.2. Titration of doxorubicin into mitochondrial lysates obtained from B1 cells (**a**), B2 cells (**b**), and B9 cells (**c**). The solid line represents the best fit of the experimental data, using a two set of site model from Microcal Origin. Relative dissociation constants were measured from ITC experiments (**d**). An overview of our findings and their potential future implications are presented (**e**). Dotted line in E represents future implications of the current findings

Since the OXPHOS complex-I is involved in DNA-damage-induced apoptosis, we hypothesize that anticancer agents such as doxorubicin bind with complex-I to initiate the apoptosis process. To confirm the specificity of doxorubicin binding to mitochondria, we performed the isothermal titration calorimetric experiments on the lysed mitochondria isolated from WT (B1), complex-I-deficient (B2), and complex-II-deficient (B9) cells. Titrations were performed using the whole mitochondrial lysate, without an isolated binding target, thus the measured binding affinity (K_d_) values are not absolute and bear significance in a relative context. Therefore, they are referred to as Kd (rel). In addition, the values of the molar ratio (*x* axis in the binding isotherm) represent arbitrary values because of lack of information on the actual concentration of the complexes (complex-I or -II) present in the mitochondrial extract. The binding isotherms representing the titration of doxorubicin with control (B1 cells) mitochondrial extract (containing both complex-I and -II) and mitochondrial extract lacking complex-II (B9 cells) are exothermic, suggesting that the binding of doxorubicin to its molecular target in the mitochondria proceeds via the evolution of heat (Figures 8a and c). Both these titration curves fit best to a two-site binding model. Binding of doxorubicin to the control mitochondrial extract appears to occur in two phases and the initial phase of binding appears to be significantly stronger [Kd(rel)~5.6 nM) than the latter phase [Kd(rel)~21 *μ*M] of binding (Figure 8d). The binding of doxorubicin, in both the phases, to the mitochondrial extract (lacking complex-II) appears to be weaker (Figures 8c and d). No discernable binding is observed when the drug is titrated against the mitochondrial extract from B2 cells lacking in complex-I (Figures 8b and d). The results of the isothermal titration calorimetry (ITC) experiment support our conclusion that DNA-damaging agent doxorubicin binds to the molecular target(s) in the complex-I. The observed moderate decrease in binding affinity of doxorubicin to the mitochondrial extract from B9 cells lacking complex-II, probably reflects that the molecular components in complex-II contribute to minor nonspecific binding of the drug.

## Discussion

Multiple anticancer agents with variant mechanisms of actions are used in the treatment of metastatic cancer including prostate and colon cancers. Some agents show greater anticancer effects than others, however, none of the current anticancer agents are sufficient to cure solid tumors. This raises the question whether anticancer agents show differential apoptotic sensitivity. This study for the first time provides effects of multiple anticancer agents including DNA-damaging agents, HDAC inhibitors, mitotic inhibitors, ER stressor, tyrosine kinase inhibitor on mitochondrial apoptosis. Although these anticancer agents induce cancer cell death, caspase activation does not always associate with mitochondria biogenesis. Etoposide, apicidin, and thapsigargin target OXPHOS complex for induction or inhibition of apoptotic cell death.

Etoposide-induced ROS production may modulate mitochondrial function causing loss of mitochondria membrane potential and leakage of proapoptotic proteins cytochrome *c*, second mitochondria-derived activator of caspases, and AIF from mitochondria.^2, 5, 6^ These proapoptotic proteins may initiate different apoptotic signaling causing demise of cancer cells. Therefore, anticancer agents such as DNA-damaging agents enhance OXPHOS function causing elevation of mitochondrial ROS, which activate inflammatory response leading to mitochondria dysfunction and apoptosis. However, whether mitochondrial ROS-mediated inflammatory response has significance in induction of apoptosis needs to be further investigated. Mitochondrial cardiolipin is required for inflammatory response^33^ and mtDNA has role in inflammatory signaling.^34^ Thus mitochondrial ROS damage cardiolipin, which collaborate with matrix-localized mtDNA to provide a platform for inflammasome activation to regulate apoptosis.

ROS have an important role in mitochondrial biogenesis^23^ and unchanged DNA-damage-induced mtDNA copy number in the presence of caspase inhibitor suggests that DNA-damage-induced ROS production was not modulated by the inhibition of caspase cascade. This notion is also supported by the fact that mitochondrial and upstream of mitochondria apoptotic signaling do not require caspase activation.^35^ MtDNA encodes 13 polypeptides critical for OXPHOS function and mtDNA copy number is regulated by multiple factors including POLG, Twinkle, and p53.^24, 36^ TP53, a tumor suppressor protein has been known to maintain mtDNA,^37, 38^ therefore, lack of p53 will ultimately lead to reduction of mtDNA copy number. Similarly, this study also showed that p53-deficiency attenuated etoposide-induced mtDNA, further indicating a possible role for p53 in OXPHOS function and mitochondrial biogenesis.

In response to stress, attenuation of damage incurred by ROS is facilitated by mitochondrial protein-folding machinery, which involves upregulation of UPR^mt^ leading to increased expression of proteases ClpP and chaperonin Hsp60. Upregulation of Hsp60 protein while decreased mRNA level suggests that increased translation of Hsps is not accompanied by enhancement of transcriptional activity. Increased accumulation of unfolded proteins, and thus UPR^mt^ can potentially compromise mitochondria function causing increased ROS accumulation and degradation of mitochondria. Therefore, ROS-mediated degradation of defective mitochondria may ultimately lead to mitochondrial biogenesis, whereas inhibition of autophagy causes accumulation of defective mitochondria, inhibition of mitochondrial proliferation, which eventually lead to decreased mtDNA levels. Indeed, the expression of POLG protein, a key regulator of mtDNA replication^25^ was reduced upon autophagy inhibition.

MtDNA content was reduced by inhibition of individual complexes, suggesting that uncoupling of respiratory complexes blocks electron transfer within OXPHOS complexes. Reduced OXPHOS complex activity may ultimately lead to moderate level of ROS causing reduction in mitochondrial biogenesis. Therefore, inhibition of OXPHOS complexes leads to reduced mtDNA. Our findings are consistent with the notion that complex-I and -III are main source for ROS production^39^ and their inhibition is expected to decrease ROS on etoposide/doxorubicin treatment.

We have identified an interesting OXPHOS-dependent apoptotic mechanism via mtDNA upregulation, and therefore, have evaluated the requirement of this pathway for the efficacy of a wide array of anticancer agents. Previous studies also suggest that cells lacking mtDNA are highly resistant to anticancer agents.^11, 40^ These studies provide key insight into the differential mechanisms of anticancer drug-induced apoptosis and lay a foundation for new effective combination therapy. Based on our findings, we describe an overview of present and potential future studies in mitochondria research (Figure 8e). DNA-damaging agents increase mtDNA/mitochondrial biogenesis through a p53-dependent and autophagy-dependent mechanism. Subsequently, this leads to complex-I-dependent ROS production and cellular apoptosis. Direct binding of doxorubicin to mitochondria in a complex-I-dependent mechanism defines a novel function of this drug class and identifies complex-I as an effective future anticancer drug target for cancer treatment. Apicidin, and sorafenib also increase mtDNA, however, further investigations are needed to define the underlying mechanisms of OXPHOS-dependent apoptosis for these two agents. Taxol, staurosporine, and thapsigargin, induce apoptosis independently of mtDNA increase. Therefore, rational combination of DNA-damaging agents with drugs of different mechanism of actions may lead to strong synergy for efficient cancer therapy. Since AMP-activated protein kinase (AMPK) activation on etoposide treatment is responsible for the induction of mitochondrial biogenesis,^41^ AMPK activators (i.e. metformin) could potentially be utilized in combination with mtDNA-independent agents (i.e. taxol) to enhance apoptosis in cancer.^42^ As metformin is relatively non-toxic, this could be an alternative to highly toxic combinational chemotherapy regimens. Together, we have defined the underlying mechanisms of OXPHOS-dependent apoptotic pathway utilized by multiple anticancer agents, which may be exploited for future anticancer strategies or development of more efficacious anticancer agents.

## Materials and Methods

### Cells and reagents

Isogenic HCT116 wild type (WT), HCT116 Bax^−/−^, HCT116 p21^−/−^ and HCT116 p53^−/−^ colon cancer cells were kindly provided by Dr. B. Vogelstein and cultured in McCoy's 5A medium supplemented with 7% FBS. Other cell lines including LNCaP were obtained from ATCC and cultured as per recommendation. All human cell lines were authenticated using the STR DNA profiling every 6 months. Chinese hamster lung fibroblasts CCL16-B1 (B1), CCL16-B2 (B2), CCL16-B9 (B9), V79-G3 (G3), V79-G7 (G7), and V79-G18 (G18) were gift from Dr. I. E. Scheffler (University of California San Diego).^28, 29, 31, 43, 44^ B2 and G18 cells lack complex-I owing to null mutations in *Ndufa1* and *Ndufb11* genes, respectively. G7 cells are impaired in initiation of the mitochondrial protein synthesis, and therefore, lack mtDNA-encoded subunits of complexes I, III, IV, and V. B1 and G3 cells are wild types for B-series and G-series mutants, respectively.

The primary antibody against heat-shock protein (Hsp) 60 (Merck Millipore, Billerica, MA, USA), Hsp10 and Hsp70 (Enzo Life Sciences, Farmingdale, NY, USA), Hsp90 (R&D System, Minneapolis, MN, USA), ClpP (Abcam, Cambridge, MA, USA), and actin (mAb; ICN) were obtained from the indicated suppliers. Secondary antibodies and ECL reagents were acquired from GE Healthcare (Pittsburgh, PA, USA). MitoTracker Green, MitoTracker Orange, CM-H_2_XRos, MitoSox, and dihydrorhodamine 123 (DHR123) were purchased from Life Technologies/Molecular Probes (Grand Island, NY, USA). The fluorogenic caspase-3 substrate DEVD-AFC and caspase-9 substrate LEHD-AFC were obtained from Enzo Life Sciences. All other chemicals were purchased from Sigma Chemical Company (St. Louis, MO, USA) unless specified otherwise.

### Whole cell lysate preparation, subcellular fractionation and Western blotting

Preparation of whole cell lysates, mitochondrial and cytosolic fractions, and western blotting were performed as previously described.^11^ The quantification of protein was carried out by micro BCA method using BSA as standard.

### Quantification of apoptosis and caspase activities measurement

Harvested cells were labeled with Trypan blue dye or with DAPI to quantify both live and dead cells. DEVDase and LEHDase activities were measured as described previously.^11^

### Annexin V and propidium iodide (PI) staining

Cells were treated with various anticancer agents or with vehicle for various time periods followed by staining with Annexin-V-Alexafluor 488/PI kit (Invitrogen, Grand Island, NY, USA) according to the manufacturer's instructions. The stained cells were analyzed by flow cytometry (LSRIIA, BD Biosciences, San Jose, CA, USA) collecting 10 000 events. Data were analyzed using WinList 3D software (Variety Software House, Topsham, ME, USA).

### Analysis of mtDNA content by real-time PCR

The mtDNA levels were quantified as described previously.^30^ Briefly, total DNA, containing both mtDNA and nuclear DNA, was isolated from cells using the ZR Genomic DNA II Kit (Zymo Research, Irvine, CA, USA). After quantification of DNA by the NanoDrop8000 (Thermo Scientific, Waltham, MA, USA), mtDNA content was determined on the Applied Biosystems 7300 Real-Time PCR system. *GAPDH* and *β-actin or ATPase 8* and *cytochrome c oxidase subunit II* (*COX II*) were used for amplification of nuclear or mtDNA, respectively. Primers for *GAPDH*, *β-actin*, *ATPase 8*, *and COX II* were used as described previously and mentioned in Supplementary Table S1.^30, 45^ The real-time PCR reaction was carried out in a total reaction volume of 10 *μ*l containing 5 *μ*l of 2 × iTaq SYBR Green Supermix with ROX (Bio-Rad, Cat no. 172-5121, Hercules, CA, USA), 10 ng of template DNA, 300 nM each of forward and reverse primers, and nuclease-free water. A melting curve analysis done at the end of amplification showed the absence of nonspecific amplification or primer dimer formation. The threshold cycle number (Ct) values for each reaction were calculated using the 7300 system SDS software (Thermo Fisher Scientific, Grand Island, NY, USA). Standard curves generated from DNA obtained from untreated LNCaP cells using 10 ng to 10 pg provided PCR efficiency based on the equation E=10^(−1/slope) −-1.^46^ Average threshold cycle number (Ct) values were obtained by amplification of *COX II* (mtDNA specific) and *β-actin (*nDNA specific). MtDNA content was determined as 2^-ΔCt, or fold difference of mtDNA from nDNA.^30, 37, 47^

### Profiler array analysis

HCT116 WT cells were treated with doxorubicin (10 *μ*M for 12 and 24 h), etoposide (10 *μ*M for 24 h) or with vehicle only for controls. Cells were harvested in TriZol and total RNA was isolated using Direct-Zol RNA miniprep kit (ZymoResearch) according to the manufacturer's instructions. After quantification of RNA by the NanoDrop8000 (Thermo Scientific), 2 *μ*g total RNA was converted to cDNA using the RT^2^ first strand synthesis kit (Qiagen, Valencia, CA, USA) according to manufacturers instructions. Equal amount of cDNAs from control and treated samples were applied to heat-shock protein array (Qiagen) and mitochondrial energy metabolism array (Qiagen) and run on ABI 7300 platform. Fold change relative to vehicle treated control was calculated following the manufacturers instructions. An average of five housekeeping genes was used to normalize the expression. For real-time PCR analysis, cDNA prepared from control and 24 h etoposide treated HCT116 WT cells were used to quantitate the expression of protein involved in UPR including C/EBP homologous protein (CHOP), CCAAT/enhancer binding protein (C/EBP), ATP-dependent ClpP protease proteolytic subunit (ClpP), chaperone DnaJ (DnaJ) or Hsp40, Hsp10, Hsp60, Hsp70, Hsp90, HSF-2 and PHAP-1 using the primer pairs listed in Supplementary Table S1.

### RNA-Seq analysis

HCT116 cells were treated with etoposide (10 *μ*M for 24 h) or vehicle only. At the end of treatment, total RNA was purified using the miRNAeasy Mini Kit (Qiagen, 217004) along with on-column digestion of DNA with RNase-Free DNase Set (Qiagen, 79254) following the manufacturers' instructions. Quality of RNA was assessed and quantified using BioAnalyzer (Agilent, G2940CA, Santa Clara, CA, USA). Equal amounts of total RNA were used to generate sequencing libraries with TruSeq RNA Sample Prep Kit v2 kit (Illumina, RS-122-2001, San Diego, CA, USA) and sequenced on an Illumina HiSeq 2500 sequencer following the manufacturer's instructions. The sequence reads (8 million) passed quality filter were mapped to the human reference genome (hg19) and ENSEMBLE annotation database using TopHat.^48^ Then the mapped reads were analyzed for differentially expressed genes using Cufflinks.^48^ Genes with a false-discovery rate ⩽0.05 were selected as significantly differentially expressed for further analysis. The sequence reads will be deposited into the National Center for Biotechnology Information Sequence Read Archive.

### Measurements of cellular ROS, and mitochondrial ROS using flow cytometry

To quantify cellular ROS, unstimulated or treated cells were washed and incubated with 5 *μ*M dihydrorhodamine 123 (DHR123) in culture medium without serum at 37 °C for 30 min according to previously described methods.^17, 49, 50^ For mitochondrial ROS measurement, we used MitoSox red as described previously.^50, 51^ Briefly, cells were suspended in growth medium without serum and were incubated with freshly prepared MitoSox red (500 nM) for 30 min at 37 °C.^17^ Data are analyzed using LSRIIA followed by WinList 3D software.

### Measurement of doxorubicin binding to mitochondria using (ITC)

All ITC experiments were performed at 25 °C using the VP-ITC (Microcal Inc., Northampton, MA, USA). Mitochondria isolated from WT (B1), complex-I-deficient (B2), and complex-II-deficient (B9) cells were suspended in 10 mM sodium phosphate buffer containing 100 mM NaCl, pH 7.2; and subjected to lysis using ultra sonication (Qsonica, LLC., Newtown, CT, USA) with an output power of 10 W for 10 times with two second on and off cycles. A stock solution of doxorubicin (200 *μ*M) was prepared in the same buffer. All samples were degassed under vacuum at 25 °C for 15 min and subjected to equilibration before titration. Lysed mitochondrial suspension was loaded into the cell and the doxorubicin was filled into the syringe. Titrations were conducted with a sequential addition of 10 *μ*l of doxorubicin to the reaction cell at an interval of 240 s for a total of 30 injections. Appropriate blank titrations were performed to subtract the background heats of dilutions from the sample titrations. All data were analyzed using the Origin software provided by Microcal Inc. The raw ITC curves were best fitted to two sets of sites binding model.

### Statistical analysis

Results are presented as mean±S.D. of data from at least three independent experiments. Statistical analysis was performed by ANOVA using Sigma Stat (Systat Software, Inc., San Jose, CA, USA).

## Abbreviations

AFC: 7-amino-4-trifluoromethyl-coumarin
AIF: apoptosis-inducing factor
AMPK: AMP-activated protein kinase
C/EBP: CCAAT/enhancer binding protein
CHOP: C/EBP homologous protein
ClpP: ATP-dependent ClpP protease proteolytic subunit
COX: II cytochrome *c* oxidase subunit II
DAPI: 4',6-diamidino-2-phenylindole
DHR123: dihydrorhodamine 123
ER: endoplasmic reticulum
HDAC: histone deacetylase
Hsp: heat-shock protein
ITC: isothermal titration calorimetry
3MA: 3-methyladenine
mtDNA: mitochondrial DNA
NAC: *N*-acetyl-L-cysteine
nDNA: nuclear DNA
OXPHOS: oxidative phosphorylation
POLG: polymerase-*γ*
ROS: reactive oxygen species
Smac: second mitochondria-derived activator of caspases
UPR: unfolded protein response
UPR^mt^: mitochondrial unfolded protein response
z-VAD-FMK: carbobenzoxy-valyl-alanyl-aspartyl-[O-methyl]- fluoromethylketone
