# Supplementary material for: Oxidative phosphorylation-dependent regulation of cancer cell apoptosis in response to anticancer agents
Source: Cell Death Dis. 2015 Nov 5;6(11):e1969–. doi: 10.1038/cddis.2015.305 (PMC4670921; doi:10.1038/cddis.2015.305)
Supplement: Supplementary Figure Legends [file cddis2015305x3.docx]

**Figure S1:** Differential sensitivity of prostate cancer cells to multiple anticancer agents. **(A and B)** LNCaP prostate cancer cells were treated with etoposide (Etop; 10 μM), taxol (TX; 30 nM), apicidin (Apic; 1 μM), sorafenib (Sor; 20 μM), staurosporine (STS; 500 nM) and thapsigargin (TG; 5 μM) for various times. Cells were labeled with annexin V/PI as per manufacturer’s instructions and early and late apoptosis were quantified by flow cytometry analysis. A minimum of 10,000 events was collected for each sample. The percentage of early apoptotic cells (stained only for annexin V) and late apoptotic cells (stained for both annexin V and PI) are plotted as bar graph. Data are mean ± SD, n=3.

**Figure S2: Anticancer agents induce caspase activation in prostate and pancreatic cancer cells.** MIA PaCa-2 pancreatic cancer cells **(A)** and PC3 prostate cancer cells **(B)** were treated with etoposide (Etop; 10 μM for 48h) or doxorubicin (Dox: 10 μM for 24h). Cells lysates were prepared to measure DEVDase (caspase-3) at excitation 400/430 nm and emission 508/520 nm using DEVD-AFC as a substrate and data are presented as fold change compared to control. Data are mean ± SD, n=3.
