# Supplementary figures and images for: Oxidative phosphorylation-dependent regulation of cancer cell apoptosis in response to anticancer agents
Source: Cell Death Dis. 2015 Nov 5;6(11):e1969–. doi: 10.1038/cddis.2015.305 (PMC4670921; doi:10.1038/cddis.2015.305)

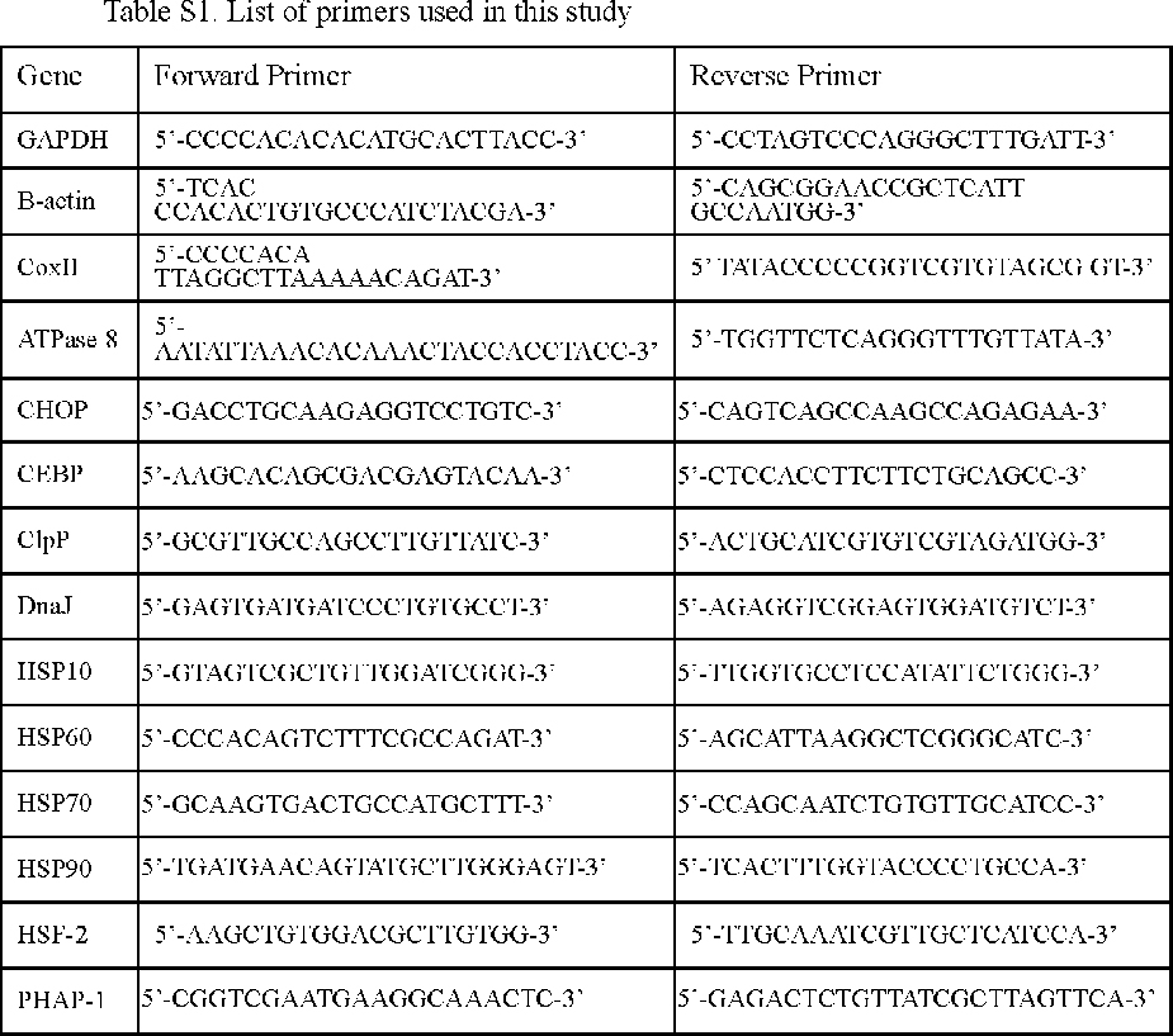

Supplement: Supplementary Table 1 [file cddis2015305x4.tif]
